# Editorial: Potential biomarkers in neurovascular disorders

**DOI:** 10.3389/fneur.2023.1186852

**Published:** 2023-04-11

**Authors:** Yuzhen Xu, Jinming Han, Wen-Jun Tu, John Zhang

**Affiliations:** ^1^The Second Affiliated Hospital of Shandong First Medical University, Taian, Shandong, China; ^2^Department of Neurology, Xuanwu Hospital, Capital Medical University, Beijing, China; ^3^Peking Union Medical College, Chinese Academy of Medical Sciences, Beijing, China; ^4^Department of Physiology and Pharmacology, Loma Linda University, Loma Linda, CA, United States

**Keywords:** neurovascular, biomarkers, peripheral blood, cerebrospinal fluid, mechanisms

Neurovascular disorders are a leading cause of disability and death in adults by affecting the arteriovenous system and blood supply. Neurological disorders accounted for 92 million disability-adjusted life-years (DALYs) in 2005 and are projected to reach 103 million worldwide by 2030 (1), serving as the leading cause of DALYs and the second leading cause of death. The annual economic burden due to neurovascular disorders is USD 765 billion in the United States and EUR 788 billion in Europe (2, 3). In view of the huge economic burden and high morbidity of neurovascular disorders, the identification of potential early biomarkers is crucial.

Ischemic stroke is the most common neurovascular disorder. He et al. found that the decreased serum transthyretin level in patients with acute ischemic stroke may serve as one major cause of intracranial atherosclerosis. Wang F.-H. et al. indicated that the decreased plasma adiponectin levels and increased visceral adipose tissue (VAT) to subcutaneous adipose tissue (SAT) ratio may be involved in intracranial atherosclerotic stenosis. Based on the report from Ding et al., Cystatin C, encoded by CST3, is a potential biomarker of atherosclerosis, with its rs13038305 and rs911119 polymorphisms independently affecting large artery atherosclerotic stroke. Furthermore, Wang A. et al. showed that the stability of atherosclerotic plaque can be affected by the non-high-density lipoprotein cholesterol (non-HDL-C)/high-density of lipoprotein cholesterol (HDL-C) ratio. The higher the ratio, the less stable the atherosclerotic plaque appears to be. The understanding of blood glucose management with intravenous thrombolysis and blood pressure management after endovascular thrombectomy (EVT) in patients with acute cerebral infarction has also been expanded by new research. Cai et al. found that patients with stroke who treated with intravenous thrombolysis can aggravate the prognosis through inflammatory response and increased blood glucose fluctuation. Zhang Q. et al. found that early blood pressure management after EVT may be important for the functional outcome of acute cerebral infarction patients with a successful recanalization.

Subarachnoid hemorrhage (SAH) is a type of hemorrhagic stroke with a high mortality and disability, accounting for 5–10% of stroke. High levels of systemic immune-inflammation index (SII) predict delayed cerebral ischemia (DCI) after SAH (Chen et al.). In addition, significantly downregulated specific plasma miRNAs, including miR-23b-3p, miR-590-5p, miR-20b-5p, miR-142-3p and miR-29b-3p, may play a role in the regulation of SAH progression (Zheng et al.).

It is estimated that there are ~50 million people living with dementia today, and this number will grow to 152 million by 2050 (4). Vascular dementia (VD) is the second largest type of dementia after Alzheimer's disease. Specifically, the new myosin hormone irisin is considered to be associated with VD and reduced serum irisin levels can predict cognitive impairment in VD patients (Zhang F. et al.). The prevalence of post-stroke cognitive impairment (PSCI) ranges from 20 to 80% (5). Gao et al. found that neuroglobin is associated with cognitive impairment after intracranial hemorrhage and low levels of neuroglobin may be used as a biomarker of cognitive impairment after intracranial hemorrhage. Recent studies regarding the involvement of gut microbiota and cognitive function have gradually emerged. Lu et al. analyzed the differences in intestinal flora between cognitive impairment group and control group, providing novel insights for subsequent research.

Moyamoya disease is a disorder characterized by narrowing and blockage of arteries in the brain, which eventually leads to ischemic stroke, hemorrhagic stroke or/and seizures. The percent amplitude of fluctuation (PerAF) can be used as an effective indicator of ocular complications and emotional complications in patients with Moyamoya disease (Li et al.). Angiogenesis and reconstruction are widely used treatments for Moyamoya disease. Caveolin-1 (Cav-1) may serve as a biomarker driving angiogenesis and collateral formation in patients with Moyamoya disease after the bypass surgery (Zhao et al.). In addition, Chen et al. showed that the TGFβ1/VEGF pathway may be a key mechanism involved in vascular remodeling in patients with Moyamoya disease.

Optic nerve and vascular lesions are important parts of neurovascular disorders in the central nervous system. Using fMRI to scan patients with optic neuritis and orbital fractures in the resting state, it was found that the amplitude of low-frequency fluctuations (ALFF) can predict abnormal functional activities at different frequencies and brain regions (Yan et al.; Kang et al.). Hu et al. used voxel-based morphometry (VBM) to analyze imaging data to investigate potential morphological changes in the brains of female patients with menopausal dry eye (DE), and analyzed the relationship between VBM and behavioral performance, suggesting that DE patients have brain changes during menopause. Abnormal spontaneous activity of the region may be the underlying pathological mechanism causing the disease. Interestingly, retinal thickness is considered as a potential biomarker for both Sjogren's syndrome (Liu et al.) and Pterygium (Wang F. et al.).

## Author contributions

YX and JH drafted the manuscript. All authors contributed to the article and approved the submitted version.
